# Discovery of a Novel MyD88 Inhibitor M20 and Its Protection Against Sepsis-Mediated Acute Lung Injury

**DOI:** 10.3389/fphar.2021.775117

**Published:** 2021-11-29

**Authors:** Jiali Song, Daoxing Chen, Yingqiao Pan, Xueqin Shi, Qian Liu, Xiaoyao Lu, Ximing Xu, Gaozhi Chen, Yuepiao Cai

**Affiliations:** ^1^ School of Pharmaceutical Sciences, Wenzhou Medical University, Wenzhou, China; ^2^ Key Laboratory of Marine Drugs of Ministry of Education, School of Medicine and Pharmacy, Ocean University of China, Qingdao, China; ^3^ Center for Innovation Marine Drug Screening & Evaluation, Pilot National Laboratory for Marine Science and Technology (Qingdao), Qingdao, China; ^4^ Marine Biomedical Research Institute of Qingdao, Qingdao, China

**Keywords:** myeloid differentiation factor 88, computer-aided drug design, virtual screening, cryptic pocket, anti-inflammatory inhibitor, acute lung injury

## Abstract

Myeloid differentiation factor 88 (MyD88) is a hub protein in the Toll-like receptor signaling pathway, which acts as a master switch for numerous inflammatory diseases, including acute lung injury (ALI). Although this protein is considered as a crucial therapeutic target, there are currently no clinically approved MyD88-targeting drugs. Based on previous literature, here we report the discovery via computer-aided drug design (CADD) of a small molecule, M20, which functions as a novel MyD88 inhibitor to efficiently relieve lipopolysaccharide-induced inflammation both *in vitro* and *in vivo.* Computational chemistry, surface plasmon resonance detection (SPR) and biological experiments demonstrated that M20 forms an important interaction with the MyD88-Toll/interleukin-1 receptor domain and thereby inhibits the protein dimerization. Taken together, this study found a MyD88 inhibitor, M20, with a novel skeleton, which provides a crucial understanding in the development and modification of MyD88 inhibitors. Meanwhile, the favorable bioactivity of the hit compound is also conducive to the treatment of acute lung injury or other more inflammatory diseases.

## Introduction

Acute lung injury (ALI) is a serious lung disease that is clinically defined as moderate or mild acute respiratory distress syndrome (ARDS). It is caused by various direct or indirect injuries of the lung parenchyma and has an approximately 40% fatality rate (Ding et al., 2020). Sepsis, the leading cause (6–42%) of ALI (Kumar, 2020), is classically activated by lipopolysaccharide (LPS) from Gram-negative pathogens (Dickson and Lehmann, 2019). It is widely known that LPS triggers inflammatory responses by activating Toll-like receptors (TLRs) (Chen et al., 2019). In the TLR signaling pathway, most of the inflammatory responses are mediated by a vital switch molecule called Myeloid differentiation factor 88 (MyD88). Currently, the main clinical treatment methods to combat lung injury diseases include drug therapy and mechanical ventilation. Although the use of protective mechanical ventilation therapy alone plays a certain role in lung protection, this treatment fails to effectively reduce the mortality of patients. Currently, anti-inflammatory drug therapy nowadays is a comprehensive treatment method for ALI/ARDS patients, which has obtained good theoretical support and preliminary research foundation. Additionally, previous studies have reported that the design of inhibitors to prevent MyD88 from self-polymerization is also a good anti-inflammatory treatment strategy (Di Padova et al., 2018).

MyD88 is a 33-kDa protein containing an N-terminal death domain and a C-terminal Toll/interleukin-1 receptor (TIR) domain, which are separated by a short intermediate domain (Chen et al., 2020). Meantime, it is also the hub protein of the TLR signaling pathway. There is emerging evidence that MyD88 can be recruited to TLR complexes as a dimer, thus affecting the signal transduction and mediating inflammation (Di Padova et al., 2018). Therefore, the use of inhibitors targeting MyD88 is considered an advanced anti-inflammatory treatment strategy. Of note, the majority of inhibitors are peptidomimetic compounds derived from BB-loop and their further modified compounds. In 2003, Loiarro et al. (2005) first developed a peptide with anti-inflammatory activity. After this, Fantò et al. (2008) designed and synthesized a series of peptidomimetic compounds using heptapeptide as a template. Then, Xie et al. (2016) reported a new skeleton of MyD88 inhibitor. Until now, no more new skeletons of MyD88 inhibitors had been reported. Currently, protein–protein interaction (PPI) is widely regarded as one of the key processes involved in the regulation of cellular mechanisms. Thus, targeting PPIs has gradually become an alternative strategy to interfere with protein activity and modify biological processes involved in the pathology of many diseases (Thabault et al., 2021). Furthermore, the strategy of inhibiting MyD88 homodimerization has also been considered to be one of the preferred directions in the development of new inhibitors (Zhang et al., 2016; Liu et al., 2018). Unfortunately, the discovery of novel MyD88 inhibitors has been progressing slowly. Hence, developing MyD88 homodimerization inhibitors for therapeutic intervention has become an urgent and challenging need.

In recent years, computer-aided drug design (CADD) has played essential roles in modern drug discovery and development (Drayman et al., 2021; Singh et al., 2021). Meanwhile, structure-based virtual screening (SBVS) is widely adopted to design and identify more bioactive compounds based on pockets in protein structures. Nevertheless, the pockets adopted in SBVS are always acquired from protein crystallization structure directly, leading to the ignorance of the dynamic properties of the target proteins. Employing molecular dynamics simulation may contribute to better understanding of the dynamic property of the protein properties and incentivize the discovery of potential cryptic pockets. This approach encourages us to go beyond the inherent limitations of screening based solely on crystallization structure to discover newer bioactive molecules.

In this study, based on the concept of inhibiting the homodimerization of the MyD88-TIR domain, 20 potential candidate compounds with novel structures were selected from a commercial library containing more than 40,000 compounds. Finally, we are very pleased to find that all of these compounds exhibit a certain anti-inflammatory activity. Of note, we preliminarily discovered a novel small-molecule inhibitor of MyD88, M20, which provides support for our predicted patterns. We conducted a deeper exploration of this compound, which will be discussed in the following sections.

## Materials and Methods

### Molecular Dynamics Simulation

All molecular dynamics (MD) simulations were conducted using the AMBER16 software package (Salomon-Ferrer et al., 2013). Before simulation, systems were prepared using the LEaP module of the AMBER16 package. The ff14SB (Maier et al., 2015) was applied to describe proteins, and the GAFF2 (Wang et al., 2004) was applied to describe small molecules. Next, each system was soaked into a rectangular box with periodic boundary conditions of TIP3P water molecules with at least 10 Å distance around the proteins or complexes. Finally, appropriate chloride or sodium ions were added to maintain the electroneutrality of the simulation system. The MD simulation was completed by the *pmemd* module in the AMBER16 software package. The entire calculation process was divided into the following seven steps: 1) imposing a restriction force of 5.0 kcal mol^−1^ Å^−2^ on the complex or the protein, and minimizing the energy of solvent molecules; 2) A restriction force of 4.0 kcal mol^−1^ Å^−2^ was applied to the main chain of the complex or protein. Minimizing the energy of the solvent molecules and the complex or protein side chains; 3) optimizing the energy of the entire system without any restraint force; 4) heating the system gradually at constant volume from 0 K to target temperature (300, 330, and 370 K) over a coupling time of 100 ps; 5) performing 100 ps simulation to accommodate solvent density; 6) removing all restrictions, and then performing another 100 ps simulation on the entire system to relax the pressure; 7) producing the simulation with the target time in the trajectory generation stage. The dynamic trajectory obtained from the simulation will be used for further studies.

The cutoff distance of the non-bond was set to 10 Å, and the particle mesh Ewald (PME) method (Sagui and Darden, 1999) was used to manage the long-range electrostatic interaction. All covalent bonds including hydrogen atoms were restricted by the SHAKE algorithm (Kräutler et al., 2001), and the integration step length of the simulation process was 2 fs. The protein structures used here were based on the X-ray structure of the MyD88-TIR domain from the Protein Data Bank (PDB code: 4DOM, https://www.rcsb.org).

### Virtual Screening Flow

The database containing the structural information of approximately 40,640 chemicals (https://enamine.net) was used for virtual screening, and the Glide module of Schrödinger suit was employed (https://www.schrodinger.com). The X-ray structure of the MyD88-TIR domain was downloaded from the Protein Data Bank (PDB code: 4DOM) for docking. The Protein Preparation module was used to preprocess the protein crystal structure. In the grid preparation process, the grid center was defined by the CA atom of residue Leu252. Then, default settings were adopted for the other settings of the binding pocket. During the docking process, the ligand was allowed to be flexible, whereas the receptor was kept as a rigid structure. The SP mode of Glide was performed to explore the appropriate binding poses in the first round of screening, and 10,000 compounds with the highest scores were selected. In the second-round screening via the XP mode of Glide, approximately 600 molecules were selected. Eventually, according to the diversity of molecules and the potential interactive mode between small molecules and the MyD88-TIR domain, 20 top-ranking molecules were selected and purchased for bioactivity assay by clustering.

### Chemicals and Reagents

Compounds were obtained from Enamine with a purity over 90% (Supplementary Table S1). We used LPS (Solarbio, Beijing, China; L8880); CCK8 assay kit (Beyotime, Shanghai, China; C0038); TNF-α and IL-6 ELISA kits (Invitrogen, Carlsbad, CA, United States ); ERK (9102S), P-ERK (9101S), P38 (8690T), P-P38 (4631S), JNK (9252T), P-JNK (4668T), MyD88 (D80F5), GAPDH (2118S), rabbit IgG (7074P2), and mouse IgG (7076P2) antibodies (CST, Shanghai, China); HA antibody (Santa Cruz, CA, United States ; sc-7392); FLAG antibody (Proteintech, Wuhan, China; 20543-1-AP); Ly6G antibody (Servicebio, Wuhan, China; GB11229); F4/80 antibody (Servicebio, Wuhan, China; GB11027); and Prime Script™ RT reagent kit (Takara, Shiga, Japan; RR047A).

### Preparation of the Compounds

The 20 selected compounds, including M20, were dissolved in dimethyl sulfoxide to prepare 10-mM stock solutions and then frozen at −20°C for further study. M20 had a purity of 98.14%. M20 was dissolved in 10% dimethyl sulfoxide and 90% corn oil for *in vivo* assay. The control group and the ALI group were given the same solvent (10% dimethyl sulfoxide and 90% corn oil).

### Cell Culture and Treatment

The extraction and cultivation methods of mouse peritoneal primary macrophages (MPMs) were as described previously (Liu et al., 2021). HEK293T cells were purchased from American Type Culture Collection (ATCC, Manassas, VA, United States ), and were cultured in DMEM (Gibco, Eggenstein, Germany) accompanied with 10% (v/v) FBS, 100 mg/ml streptomycin, and 100 U/mL penicillin G.

### ELISA

After incubation with the compounds for 45 min, MPMs were then incubated with LPS (500 ng/ml). Compounds and LPS were incubated with cells in the next twenty-four hours. After that, the sample supernatants were collected and centrifuged at 24,000*g* for 10 min at 4°C. Following the manufacturer’s instructions, the TNF-α and IL-6 ELISA kits were used to detect the secretion of pro-inflammatory cytokines in the supernatant.

### Quantitative Real-Time PCR

Total RNA from MPMs or lung tissues was extracted by TRIzol reagent (Invitrogen, Carlsbad, CA, United States ). The RNA concentration was measured at a ratio of 260/280 nm, and the samples with A260/A280 in the range of 1.8–2.0 were allowed for further study. Then, reverse transcription was carried out using the Prime Script™ RT reagent kit. Next, SYBR Green Super mix (Bio-Rad, Hercules, CA, United States ) was used for amplification. The primer sequences of the genes used are shown in Supplementary Table S2.

### Western Blot Analysis

Proteins were extracted from cells or lung tissue using lysis buffers (Boster, Wuhan, China) with pre-added protein phosphatase inhibitors (Applygen, Beijing, China) according to the manufacturer’s instructions. The tissues were homogenized into small pieces before lysis so that the protein could be extracted fully in the lysis solution. After the lysis was completed, protein levels were determined by the BCA assay kit (Beyotime, Shanghai, China). Next, the prepared samples were separated by 10% SDS-PAGE and electro-transferred to polyvinylidene difluoride membranes. After this, the membranes were blocked with 10% milk (BD, Franklin Lakes, NJ, United States ) and then exposed to primary antibodies at 4°C overnight. The next day, the membranes were incubated with the corresponding secondary antibody. The immune complexes were visualized by enhanced chemiluminescence reagent (Bio-Rad, Hercules, CA, United States ).

### Animals

Eight-week-old male C57BL/6J mice with a weight of 18–22 g were obtained from the Animal Center of Wenzhou Medical University and were given access to a chow diet and water *ad libitum*. Before the experiment, the mice were housed in a pathogen-free animal facility with the temperature maintained at 22–24°C under a 12-h light/dark cycle. All animal experiments were approved by the Wenzhou Medical College Animal Policy and Welfare Committee and were performed in accordance with the Code of Ethics of the World Medical Association.

These mice were randomly allotted to four groups: control group (*n* = 6); ALI group (*n* = 6); 20 mg/kg M20 pre-treated group (*n* = 6); and 40 mg/kg M20 pre-treated group (*n* = 6).

The mice were injected, intravenously (iv) through the tail vein, with LPS (15 mg/kg, 0.9% saline) or an equal volume of 0.9% saline. Mice were pre-administered with M20 intraperitoneally (ip) 6 h and 30 min before LPS injection. Six hours later, the mice were sacrificed. Finally, bronchoalveolar lavage fluid (BALF), blood samples and lung tissues were collected from the mice and stored in a refrigerator at −80°C.

### Histological Assay

The lung tissues were fixed in 4% paraformaldehyde for paraffin embedding and histopathological analysis. These tissue specimens were sectioned to 4-μm-thick slices and they underwent hematoxylin and eosin staining (H&E detection kit) and immunohistochemistry (IHC) staining in accordance with the kit instructions. Images were obtained with a Nikon microscope.

### BALF Analysis

We inserted a sterilized pipette tip into the trachea and then injected ice-cold PBS into the lungs. A 1-ml sample of BALF was obtained and centrifuged at 24,000 *g* for 15 min at 4°C. The supernatant was then taken to detect the level of inflammatory factors and the protein concentration of the lung tissue, while the lower sediment was resuspended in 30 μl PBS to determine the total number of cells in BALF (Liu et al., 2021).

### Surface Plasmon Resonance Detection

A protein interaction array system (SPR Instrument, Biacore T200, GE, Connecticut, United States ) was used to detect the binding between the protein and the molecule. We used 1 × PBS (5% DMSO, 0.5% Tween 20) for sample dilution and analysis. Both 0.4 M N-ethyl-N-[3-dimethylaminopropyl]-carbodiimide and 0.1 M N-hydroxysuccinimide were used to activate the CM7 protein chip. The recombinant MyD88 protein was dissolved in 10 mM sodium acetate buffer without Tris and other amino-containing compounds. The acid salt was cured to allow for the excess N-hydroxy succinimide ester to block unreacted active sites. To detect the binding between MyD88 protein and M20, the compound M20 with different concentrations (0 μM, 1.95 μM, 3.91 μM, 7.81 μM, 15.6 μM, 31.3 μM, 62.5 μM, 125 Μm, and 250 μM), were dissolved in 1 × PBS (5% DMSO, 0.5% Tween 20) buffer and passed through the sensor chip at a flow rate of 60 μl/min. The response curves of the samples with different concentrations were all displayed after subtracting the reference flow cell. The Biacore T200 instrument analysis software was used to subtract the control value and to perform the kinetic fitting mathematical model analysis.

### Co-Immunoprecipitation Assay

After protein quantification, HA antibody was added and incubated with the samples at 4°C overnight. The next day, protein A+ G agarose beads were added and incubated for 4 h before collecting protein. The agarose beads were washed with PBS and then boiled in the sample buffer. The sample was used to detect FLAG epitopes of the dimerization of MyD88 and the density of immune reaction bands was analyzed using ImageJ software.

### Statistical Analysis

All experiments followed the principle of randomization and blindness. Data were expressed as the mean ± standard error of mean (SEM) as indicated. Different groups were compared with Student’s *t* test. Statistical analysis was performed with GraphPad Prism software (GraphPad Prism Software, San Diego, CA, United States ). *p* values <0.05 were considered statistically significant.

## Results

### Study of MyD88-TIR Stability

Many of the previous MyD88 inhibitors were designed based on BB-loop peptidomimetics. However, we wanted to explore potential druggable regions in MyD88-TIR domain other than the BB-loop. On the basis of previous studies and the protein itself, it is possible that we may be able to find new inhibitors of MyD88. Therefore, here we conducted an in-depth analysis of the MyD88 protein structure (PDB code: 4DOM).

**FIGURE 1:**
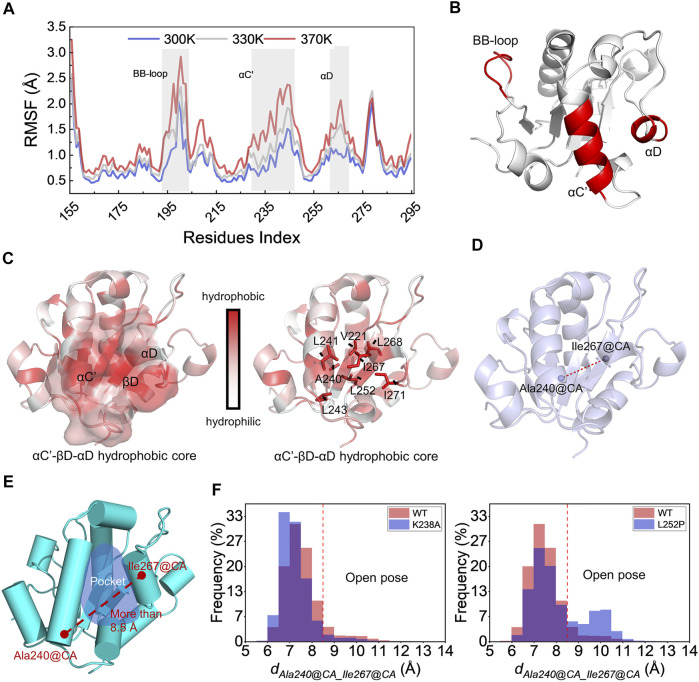
Study of MyD88-TIR stability and identification of potential druggable site. **(A)** Root mean square fluctuations of the CA atoms for the wild-type MyD88-TIR monomer MD simulation at the temperatures of 300, 330, and 370 K. **(B)** The three-dimensional locations of BB-loop, αC′, and αD motif. **(C)** The hydrophobicity analysis, red represented hydrophobicity and white represented hydrophilicity. **(D)** Schematic diagram of the distance between Ala240@CA and Ile267@CA (*d*_*Ala240@CA_Ile267@CA*_) on the MyD88-TIR domain. **(E)** The “Open pose” which was found in MD simulation. **(F)** Statistical results of *d*_*Ala240@CA_Ile267@CA*_ changes during MD simulation.

Proteins are in an irregular state of motion in most organisms. They often have highly flexible regions that can directly or indirectly determine the PPIs (Yu and Huang, 2014). As for the MyD88-TIR domain, identifying its flexible region is beneficial to understand the dynamic characteristics and polymerization mode. To capture the MyD88-TIR flexible region, in MD simulations, the protein was placed at the temperatures of 300, 330, and 370 K for 50 ns each. MD simulations were performed at higher temperatures in order to reveal more information related to protein unfolding (Yu et al., 2017). As shown in Figures 1A,B, the BB-loop (193–204 aa), αC' (231–246 aa), and αD (262–270 aa) domains were more flexible than other regions. This indicated that the BB-loop, αC′ and αD have abnormal dynamic properties.

There has been a large amount of research on the effect of BB-loop motif on the polymerization of the TIR domain (Loiarro et al., 2005; Jiang et al., 2006; Ohnishi et al., 2009; Olson et al., 2015; Clabbers et al., 2021). Many of them have made successive efforts, but mostly focused on the realm of peptides derived from BB-loop and their derivatives. Hence, here we mainly focused on the αC′ and αD motifs. As shown in Figure 1C, the protein is colored by residue hydrophobicity. Motifs αC′ and αD are adjacent, and αC′ touches αD with a hydrophobic interaction. It is worth noting that αC′, αD, and βD can form a hydrophobic core here. Figure 1C shows that the residue Leu252 of βD further connects αC′ and αD via hydrophobic interaction. This finding is consistent with the previously reported L252P mutation (Zhan et al., 2016), which also affects the dynamic status of TIR. Combined with the standpoint reported by previous studies, it was found that L252P mutation can promote MyD88 polymerization (Ngo et al., 2011; Loiarro et al., 2013; Vyncke et al., 2016). A possible mechanism for the L252P mutation involves its effect on the αC′–βD–αD hydrophobic core.

### Identification of a Potential Druggable Site

To verify the above hypothesis, long-time MD simulations were used to explore changes in the αC′–βD–αD hydrophobic core. Wild-type TIR, polymerization-inhibitable mutant K238A (Vyncke et al., 2016; Ve et al., 2017), and polymerization-promotable mutant L252P were subjected to 300 ns MD simulation. The stability of the αC′–βD–αD hydrophobic core was reflected by the changes in the distance between the CA atoms on the residue Ala240 and the CA atoms on residue Ile267 (*d*_*Ala240@CA_Ile267@CA*_) during the MD simulation (Figure 1D). After this calculation, we observed that the hydrophobic core was destroyed, and a hydrophobic pocket was formed when *d*_*Ala240@CA_Ile267@CA*_ was greater than 8.5 Å (Figure 1E). All the results of MD systems are shown in Figure 1F. The *d*_*Ala240@CA_Ile267@CA*_ of WT and the K238A mutant were likely to be less than 8.5 Å during the long-time MD simulation. Especially for K238A, there was almost no conformation with *d*_*Ala240@CA_Ile267@CA*_ greater than 8.5 Å. In contrast, the L252P mutant had a relatively high probability of sampling conformation with *d*_*Ala240@CA_Ile267@CA*_ greater than 8.5 Å. This means that L252P could destroy the hydrophobic core, i.e., promote the separation of αC′ and αD. This preferred conformation brought about by the L252P mutation is very likely to be the polymer conformation, which we called “Open pose” here. The surface of αC′ and αD in the “Open pose” might directly participate in the MyD88-TIR aggregation.

In summary, based on the in-depth analysis of the MyD88 protein structure, we identified flexible regions other than the BB-loop and further discovered the hydrophobic core. The destruction of the hydrophobic core can form a hydrophobic pocket, which is a potential druggable site to discover inhibitors that block the polymerization of MyD88-TIR domain. Next, we carried out the following virtual screening.

### Preliminary Discovery of a Small Molecular Inhibitor M20, Which Targets the MyD88-TIR Domain

**FIGURE 2:**
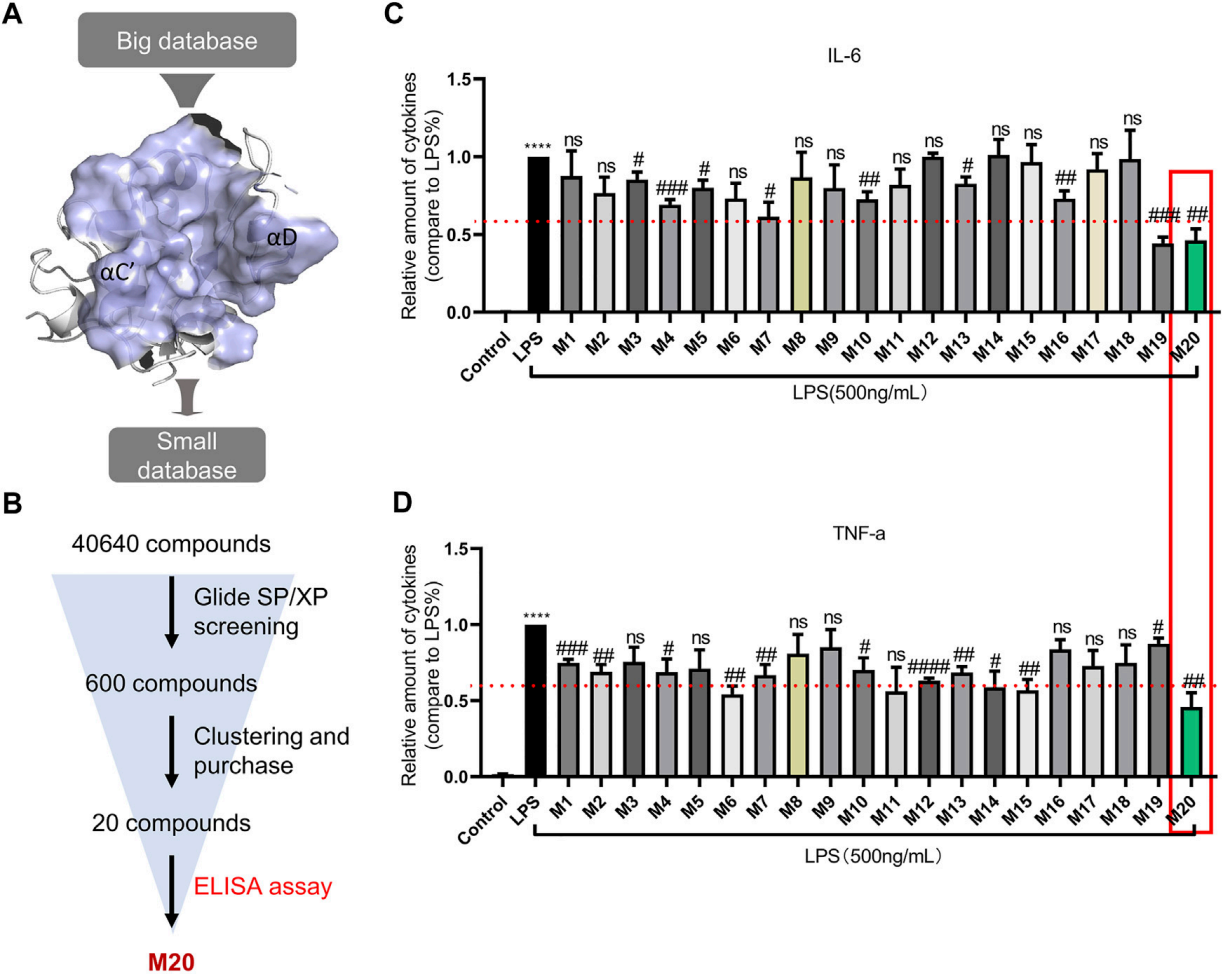
Preliminary discovery of a small molecular inhibitor M20, which targets the MyD88-TIR domain. **(A)** The druggable pocket of “Open pose” for structure-based virtual screening. **(B)** Workflow of screening for new MyD88 inhibitors. The concentrations of IL-6 **(C)** and TNF-α **(D)** in supernatants from MPMs that were pre-treated with 10-μM dose of compounds, followed by stimulation with 500 ng/ml LPS for 24 h and were detected by ELISA. The data were expressed as the mean ± SEM as indicated. Different groups were compared with Student’s *t* test. It was utilized for the statistical analysis, and significant differences were indicated as ^*^ or ^#^. ^****^ *p* < 0.0001 vs the control group; ^#^ *p* < 0.05, ^##^ *p* < 0.01, ^###^ *p* < 0.005, and ^####^ *p* < 0.0001 vs the LPS alone group.

The employment of structure-based drug design is a distinctive approach in discovering new inhibitors or drug molecules in a time-saving and cost-effective manner (Singh et al., 2021). Therefore, virtual screening was employed to sift the database of 40,640 small molecules from the Enamine library and discover those potentially inhibiting MyD88-TIR homodimerization. As shown in Figure 2A, the “Open pose” was used for structure-based virtual screening. After docking via the Glide SP/XP methods, 600 binding poses of compounds were produced with MyD88-TIR domain. All the compounds with a docking score greater than the cutoff score of −5 kcal/mol were selected for scaffold clustering through the Canvas module.

Twenty top-ranking molecules, each of a different scaffold, were selected for purchase and assay (Supplementary Table S1; Figure 2A,B). Compounds that exhibited pro-inflammatory cytokine inhibition rate greater than 40% at 10 μM were selected for further biological activity assay. In this round of screening, the compound M20 stood out with approximately 50% inhibition rates both for TNF-α and IL-6, whereas M4, M7, M10, M13, and M19 failed to achieve more effective inhibition rates on the two indexes (Figure 2C,D).

**FIGURE 3:**
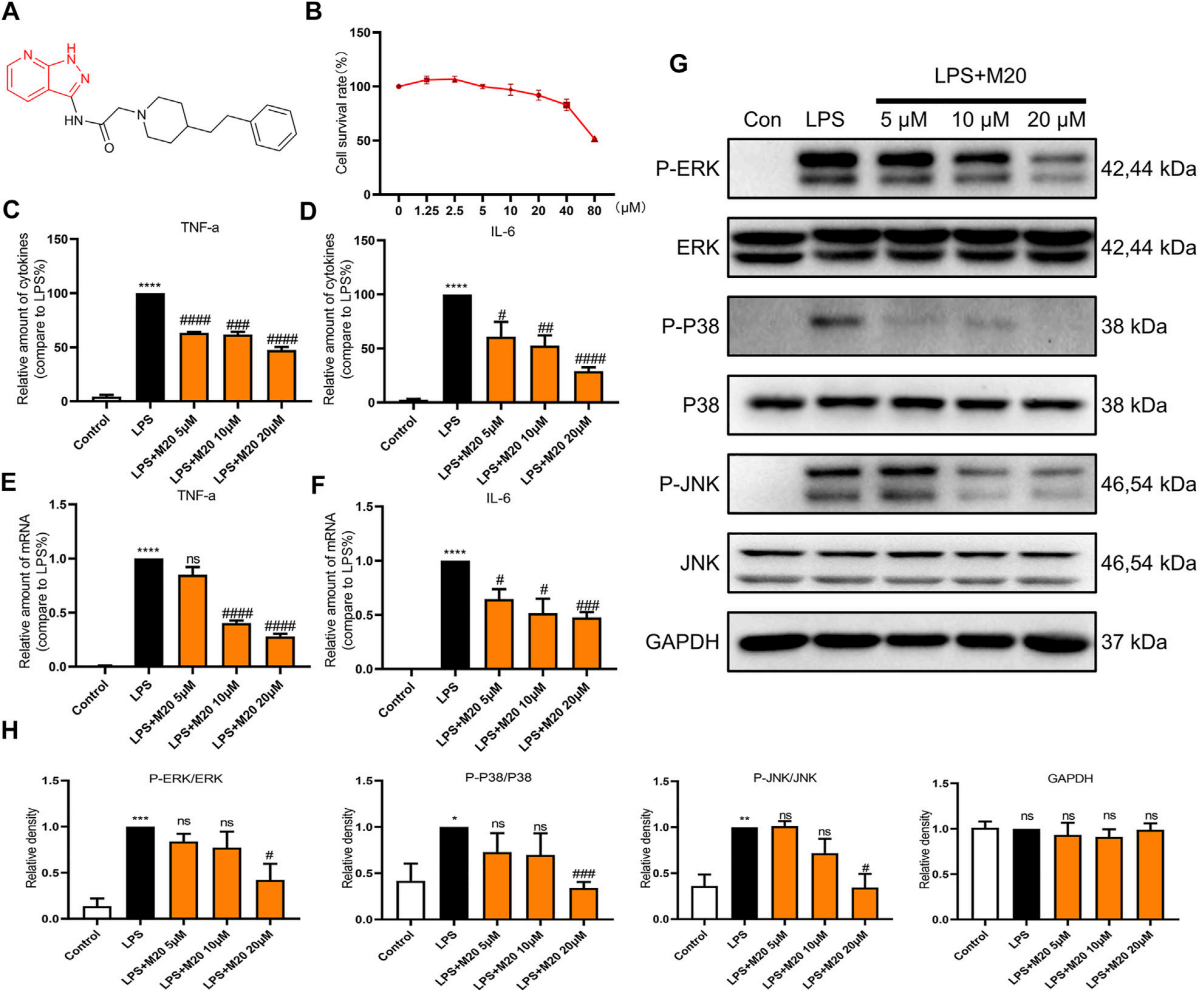
M20 is a potent compound that inhibits LPS-induced inflammation *in vitro*. **(A)** The structure of M20. **(B)** The cell survival rates of M20 (1.25, 2.5, 5, 10, 20, 40, and 80 μM) in MPMs. The concentrations of TNF-α **(C)** and IL-6 **(D)** in supernatants from MPMs that were measured by ELISA. The TNF-α **(E)** and IL-6 **(F)** mRNA levels of MPMs that were determined by RT-qPCR. **(G)** The protein levels of the P-ERK, P-P38 and P-JNK that were detected by western blot. **(H)** Densitometric quantification was presented as the mean ± SEM (*n* = 3) in the lower panel. Data were expressed as the mean ± SEM as indicated. Different groups were compared with Student’s *t* test. It was utilized for the statistical analysis, and significant differences were indicated as ^*^ or ^#^. ^****^ *p* < 0.0001 vs the control group; ^#^ *p* < 0.05, ^##^ *p* < 0.01, ^###^ *p* < 0.005, and ^####^ *p* < 0.0001 vs the LPS alone group.

Thus, M20 was selected as a compound with great potential for further biological activity evaluation. To provide a potential treatment for MyD88-driven inflammation, we conducted a deeper activity study on pyrazolo[3,4-b] pyridine analog M20. The chemical structure of M20 is shown in Figure 3A.

### M20 Is a Potent Compound that Inhibits LPS-Induced Inflammation in Vitro

Previous studies have confirmed that LPS mainly activates the Toll-like receptor 4 (TLR4) signaling pathway (Lim and Staudt, 2013; Chen et al., 2020). Then, the intracellular TIR domain recruits MyD88, induces a signal cascade, promotes inflammation, and produces pro-inflammatory cytokines such as TNF-α, IL-6, and IL-1β. To avoid toxic effects, we first evaluated the effect of the candidate compound M20 on cell survival rates. The CCK-8 assay results showed that the survival rate of M20– pre-treated MPMs under 20 μM concentration was greater than 80%, which indicated the low toxicity of the compound (Figure 3B).

As the secretion of pro-inflammatory cytokines is the most intuitive indicator of inflammation, ELISA assay was first carried out to evaluate the function of M20 in LPS-induced MPMs. As shown in Figures 3C,D, the M20–pre-treated group showed the prominent inhibition of TNF-α and IL-6, two classical pro-inflammatory cytokines observed in the inflammatory response. The corresponding changes in mRNA levels of TNF-α and IL-6 were also seen during the LPS stimulation periods (Figure 3E–F). M20 downregulated the expression levels of the two inflammatory factors in a dose-dependent manner. At 20 μM, it exhibited favorable inhibition capability (TNF-α > 70%, IL-6 > 50%).

In addition, in order to better comprehend whether M20 would affect signal transduction under LPS stimulation, we further examined its performance in the phosphorylation of key proteins (ERK, P38, and JNK) in the MAPK signaling pathway since MyD88 is upstream of the classical inflammation pathway MAPK. Treatment of LPS-induced MPMs with M20 for 1 h resulted in a dose-dependent inhibition of ERK phosphorylation (Figures 3G,H), while the total amount of the target protein remained unchanged. The same decreasing trends were also observed in the phosphorylation levels of P38 and JNK (Figures 3G,H).

Collectively, these findings support the conclusion that M20 is a potent compound that can significantly mitigate LPS-induced inflammation *in vitro*.

### M20 Alleviates Sepsis-Induced ALI in Vivo

**FIGURE 4:**
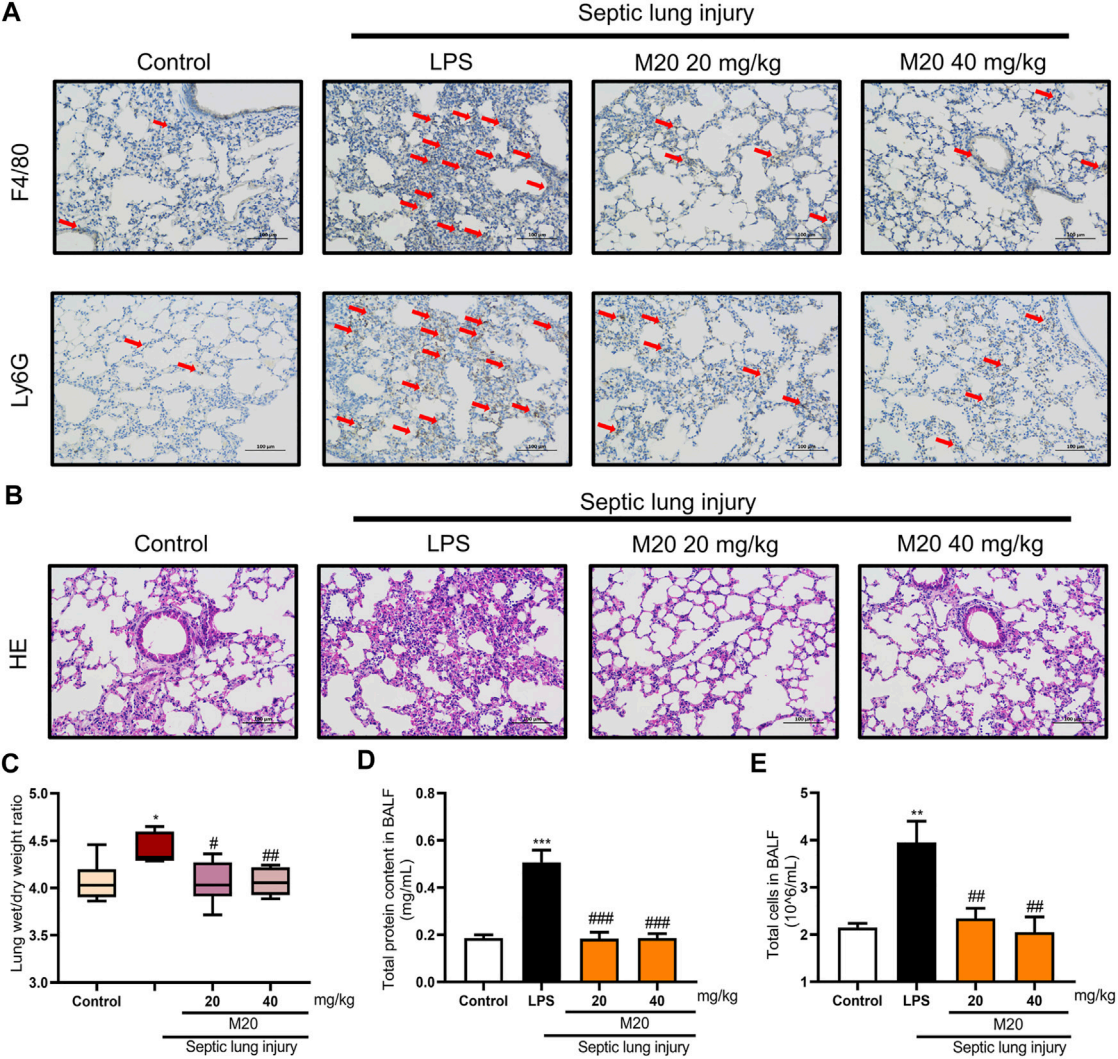
M20 alleviates sepsis-induced ALI *in vivo*. **(A)** IHC staining was used to observe the distribution of neutrophil positive spots. **(B)** H&E staining was used to observe histopathological changes. The lung wet/dry weight ratio **(C)** and total protein content in BALF **(D)** were measured to evaluate pulmonary edema. **(E)** The total cells in BALF were measured to evaluate inflammatory infiltration. The data were presented as the mean ± SEM (*n =* 6). Different groups were compared with Student’s *t* test. It was utilized for the statistical analysis, and significant differences were indicated as ^*^ or ^#^. ^****^ *p* < 0.0001 vs the control group; ^#^ *p* < 0.05, ^##^ *p* < 0.01, ^###^ *p* < 0.005, and ^####^ *p* < 0.0001 vs the Septic lung injury alone group.

To better evaluate the anti-inflammatory activity of M20 *in vivo*, we used the sepsis-induced ALI mice model to investigate the therapeutic capability of it. When ALI occurs, type II alveolar epithelial cells are extensively damaged, leading to decreased synthesis and activity of alveolar surfactant. These changes result in damage to the respiratory membrane, followed by alveolar collapse and pulmonary edema (Yao et al., 2017). Pathological analysis was first carried out to characterize the pathohistological appearance of the lung, which is considered one of the important phenotypes of the ALI pattern. As shown in Figures 4A,B, LPS alone led to obvious destruction of the alveolar structure and induced appearance of emphysema in connective tissue. However, when M20 was applied, the signs of lung injury were significantly alleviated (Figure 4A,B). To assess neutrophil infiltration, we detected Ly6G and F4/80—two classic neutrophils. Neutrophils, observed as brown dots in the lung interstitial area, increased significantly in ALI mice; however, this increase was markedly mitigated by the pre-treatment with M20 (Figure 4A).

Pulmonary edema is another vital indicator of ALI, which can be measured by the wet/dry ratio of the lung tissues. As shown in Figures 4C,D, the wet/dry ratio of the lung tissues in the ALI group was increased, and it was accompanied by a significant increase in BALF protein content as well. In contrast, M20 treatment reversed the pulmonary edema. Next, we further measured the protein concentration in the alveolar lavage; it was found that the M20–pre-treated group effectively reduced the damage to the pulmonary microvascular barrier (Figure 4E).

**FIGURE 5:**
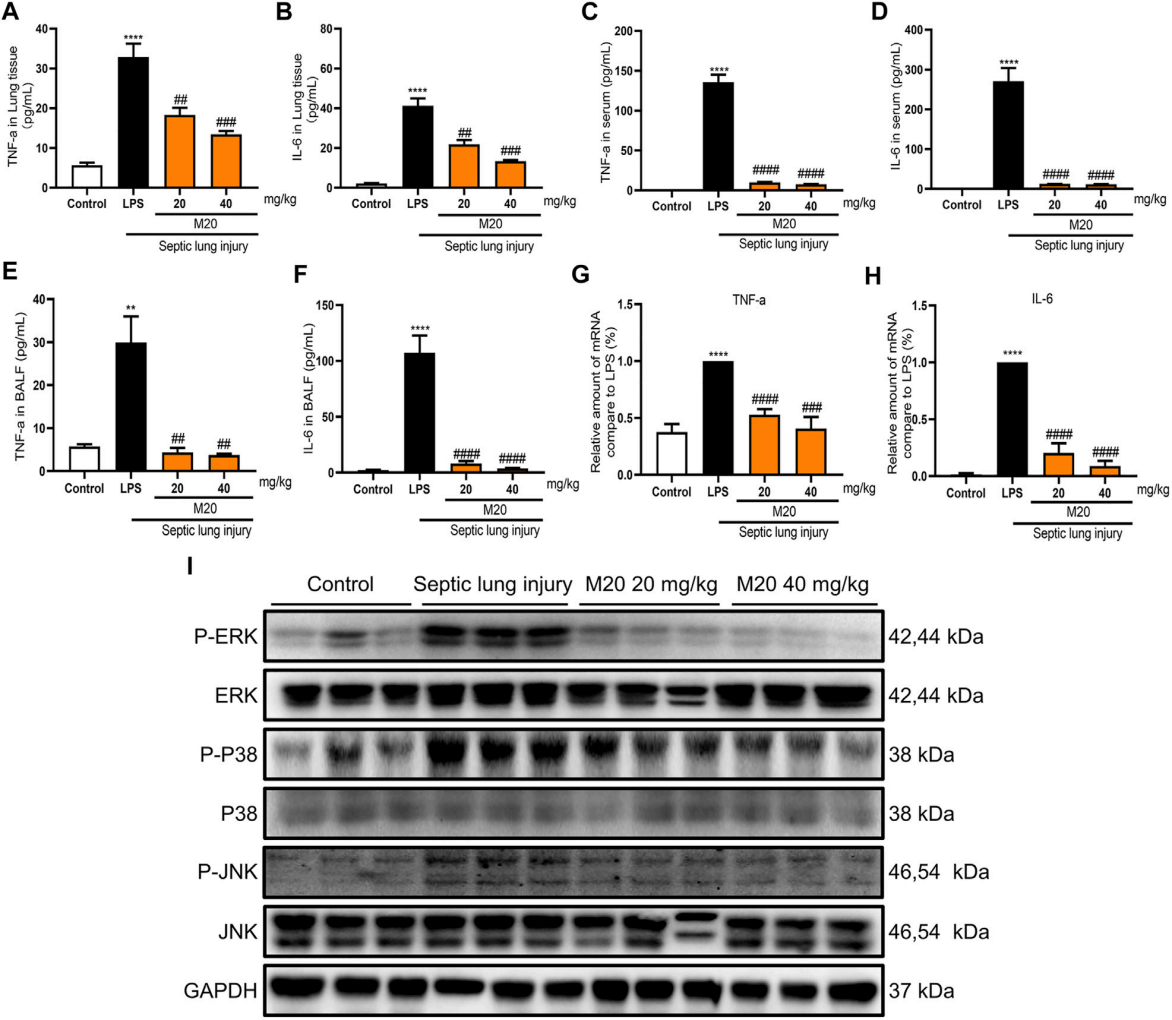
M20 alleviates sepsis-induced ALI *in vivo*. The release levels of TNF-α **(A)** and IL-6 **(B)** in lung tissues were measured by ELISA (*n* = 6). The release levels of TNF-α **(C)** and IL-6 **(D)** in serum were measured by ELISA (*n* = 6). The release levels of TNF-α **(E)** and IL-6 **(F)** in BALF were measured by ELISA(*n* = 6). The mRNA levels of TNF-α **(G)** and IL-6 **(H)** in lung tissues were measured by RT-qPCR (*n* = 6). **(I)** The protein levels of the P-ERK, P-P38 and P-JNK in lung tissues that were detected by western blot. The data were expressed as the mean ± SEM as indicated. Different groups were compared with Student’s *t* test. It was utilized for the statistical analysis, and significant differences were indicated as ^*^ or ^#^. ^****^ *p* < 0.0001 vs the control group; ^#^ *p* < 0.05, ^##^ *p* < 0.01, ^###^ *p* < 0.005, and ^####^ *p* < 0.0001 vs the Septic lung injury alone group.

Additionally, while detecting the pro-inflammatory cytokines of lung tissue homogenate supernatant, serum, and alveolar lavage fluid, we found that when challenged with a high dose of LPS, the levels of pro-inflammatory cytokines were elevated in ALI mice (Figures 5A–F). Moreover, the levels of the two inflammatory factors in the ALI–alone group were higher than those in the control group, which demonstrated the success of our modeling. What motivated us most was that the M20–pre-treated group successfully inhibited the release of pro-inflammatory cytokines with more than 90% inhibition rates when compared with the ALI-alone group (Figures 5C,D). Subsequently, we produced further evidence that this compound significantly reversed inflammation in the lungs of ALI mice by measuring the levels of activated ERK, P38, and JNK protein. The western blot results showed that 6 h of sepsis promoted the MAPK signaling pathway activation; however, the M20–pre-treated group showed a dose-dependent mitigation effect (Figure 5I). To further investigate the anti-inflammatory effect of M20, we detected the level of inflammatory gene fluctuations from the mice lungs. Compared with the ALI model group, the gene expression levels of TNF-α and IL-6 in the M20–pre-treated group were reduced in a dose-dependent manner (Figures 5G,H). ALI can cause peripheral neutrophils, macrophages, and other immune cells to infiltrate and accumulate in lung tissues, whereas M20 significantly improves acute pulmonary edema and the secretion of inflammatory factors, eventually preventing the trigger of the “inflammation waterfall” cascade effect.

In summary, all of the above results further provide us with strong evidence that M20 is a promising candidate with a novel chemical scaffold in pre-treatment of ALI and potentially other inflammatory injuries. Next, we explored the stability of the MyD88/M20 complex and the potential binding mode.

### Explore the Binding Mode of M20 and MyD88

**FIGURE 6:**
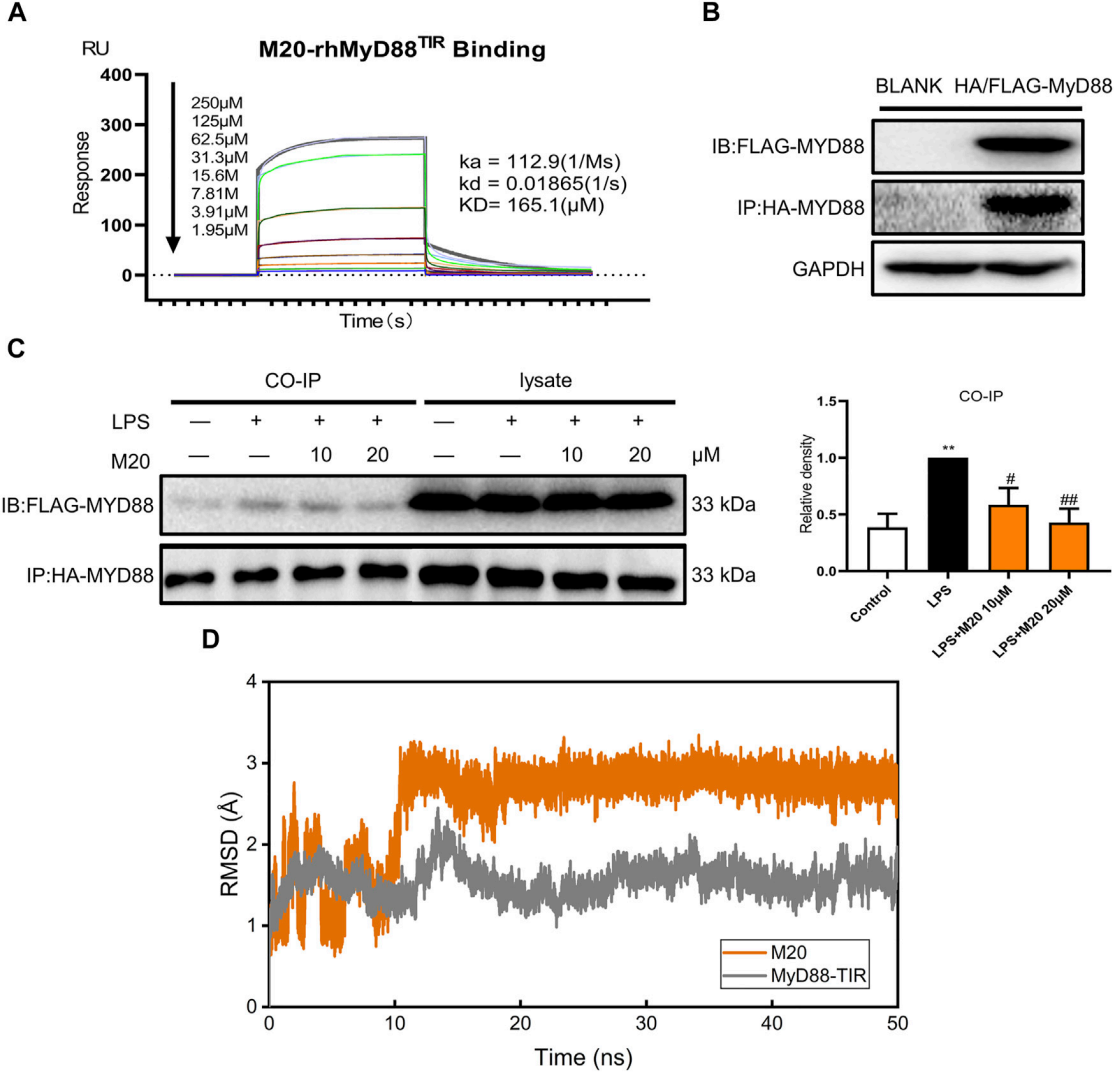
Explore the binding mode of M20 and MyD88. **(A)** SPR shows direct interactions between M20 and MyD88 in a concentration-dependent manner. **(B)** The transfection efficacy of HEK293T cells that were cotransfected with HA-MyD88 and FLAG-MyD88 for 24 h. **(C)** The effect of M20 in inhibiting MyD88 dimerization. Densitometric quantification was presented as the mean ± SEM (*n* = 3) in the right panel. **(D)** Root mean square deviation of the CA atoms in MyD88-TIR domain (gray) and heavy atoms in M20 (orange). The data were expressed as the mean ± SEM as indicated. Different groups were compared with Student’s *t* test. It was utilized for the statistical analysis, and significant differences were indicated as * or #. ^****^ *p* < 0.0001 vs the control group; ^#^ *p* < 0.05, ^##^ *p* < 0.01, ^###^ *p* < 0.005, and ^####^ *p* < 0.0001 vs the LPS alone group.

Previous studies have proven that M20 is a bioactive molecule with great potential, but it remains unknown whether M20 could inhibit MyD88 homodimerization and thereby inhibit inflammation. Whether M20 could truly conform to the initial concept of previous modeling and screening predictions, instead of merely exhibiting anti-inflammatory activity, was still remains a mystery. Thus, SPR was used to further investigate the binding of M20 with MyD88. As we can see, M20 showed a relatively optimal binding affinity toward the MyD88 protein in a dose-dependent manner (*K*_*D*_ = 165.1 μM, Figure 6A). To better confirm that the immunomodulatory function of M20 attributed to the inhibition of MyD88 homodimerization, co-immunoprecipitation was used to study the interactions of tag-proteins *in vitro*. Two plasmids encoding HA-MyD88 and FLAG-MyD88 were co-transfected into HEK-293T cells using Liposome 3,000. Western blotting showed that FLAG-MyD88 and HA-MyD88 were effectively co-expressed in HEK293T cells after 24 h (Figure 6B). Then, the transfected cells were incubated with the compound M20 for 1 h. Western blot analysis was performed 15 min after LPS induction. The results showed that M20 inhibited the formation of the MyD88-TIR domain homodimer (Figure 6C). Once the binary complex of M20 and MyD88 is formed, it interferes with the recruitment of IRAK2/IRAK4 (Sun et al., 2016) into the active site of MyD88, thereby resulting in the inability to activate the MAPK signaling pathway.

**FIGURE 7:**
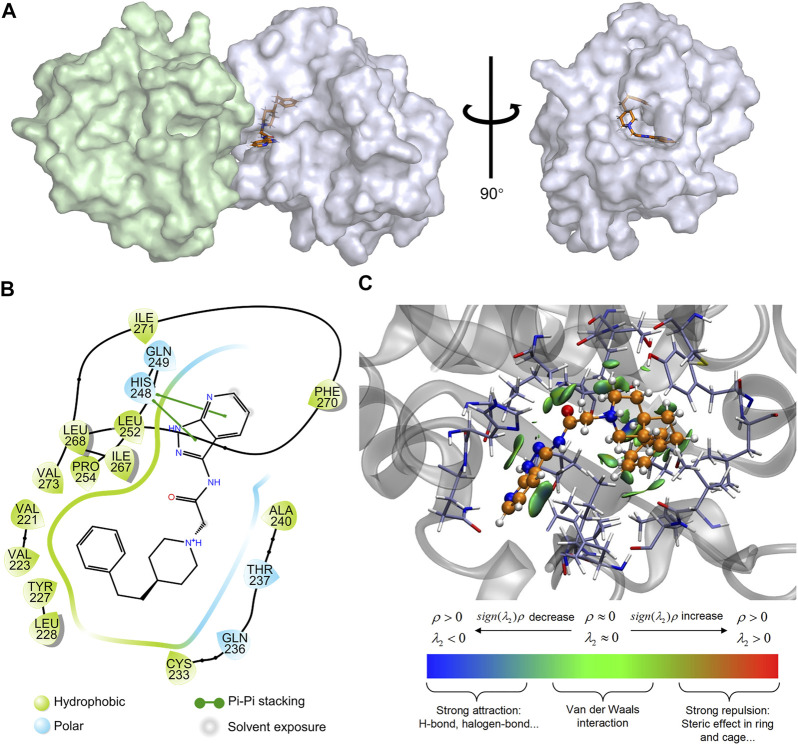
Prediction of the binding mechanism of M20 and MyD88. **(A)** The prediction of the binding mode of M20 with MyD88. M20 was depicted as orange licorice, the left region of MyD88 oligomer was colored in palegreen and the right region was colored in bluewhite. **(B)** The 2D-binding mode of M20 with MyD88-TIR domain. **(C)** The 3D-binding mode of M20 with MyD88-TIR domain with the isosurfaces of IGM and colorbar. M20 was colored in orange, and the surrounding residues of the binding pockets were colored in iceblue. The backbone of the receptor was depicted as a gray cartoon.

Additionally, to further investigate the binding mode of M20 with the MyD88-TIR domain, a 50 ns MD simulation under 300 K was carried out, and the docking outputs were adopted as the initial structure in this calculation. The RMSD plot of heavy atoms in M20 experienced fluctuation initially until 10 ns and then stabilized (Figure 6D). In addition, considering the stable RMSD fluctuation in the last 40 ns of the simulation, the last snapshot of the simulation trajectory was chosen as the binding mode of M20 with the MyD88-TIR domain. The pocket diagrams of M20 and MyD88 help in predicting the state of M20 in the protein, which lies in a suitable position in the large hydrophobic binding pocket formed by αC′–βD–αD (Figure 7A). The 2D-demonstrated binding mode (Figure 7B) showed a wide hydrophobic interaction surrounding M20 with some polar interactions. For visualization of the structure and its interactions, we conducted an Independent Gradient Model (IGM) analysis (Lefebvre et al., 2017) by Multiwfn (Lu and Chen, 2012) and 3D-visualized the isosurfaces and structure by VMD (Humphrey et al., 1996). The δg^inter^ isosurfaces (Figure 7C) exhibited that the main interaction of M20 with the MyD88-TIR domain is van der Waals interactions that occurred without strong repulsions between our compound and residues.

Apparently, the van der Waals interactions around M20 make it stably bind to the MyD88-TIR domain. With a relatively small structure, M20 can be flexibly embedded into the hydrophobic pocket, thereby changing the protein morphology and promoting MyD88 into a low-activity state. Eventually, the compound occupied most of the surface of αC′ and αD on the “Open pose” and finally achieved the purpose of inhibiting homodimerization. These results above indicated that the preferred compound, M20, could stably bind to the target protein MyD88 and owns the capability of inhibiting protein self-aggregation. At the same time, the good bioactivity of M20 demonstrated that the MyD88-targeted drug design ideas based on virtual screening to exploit more novel MyD88 inhibitors are advisable and favorable.

## Discussion

ALI is a severe clinical syndrome, and its common manifestation is diffuse pulmonary edema. Usually, it is characterized by an overwhelming inflammatory response leading to excessive damage of the alveolar epithelium. Among various causes of ALI, the most common is septic pneumonia caused by LPS. In LPS-induced TLR4 signaling pathway, MyD88 is a typical partner and plays an adaptor protein role in regulating the inflammatory response (Chen et al., 2020). The concept of combating with the TLR and interleukin receptor signaling, demonstrates that MyD88 inhibitors may have enormous potential in the treatment of acute and chronic respiratory diseases (Di Padova et al., 2018). Therefore, developing new types of MyD88 inhibitors is particularly significant for the treatment of ALI.

In recent years, researches on MyD88 inhibitors have mostly focused on the BB-loop, whereas from the surface of αC′ and αD in the “Open pose”, we discovered a druggable pocket based on these abnormal dynamic properties as well. Currently, studies have shown the standpoint that L252P mutation could promote the polymerization of MyD88 (Ngo et al., 2011; Loiarro et al., 2013; Vyncke et al., 2016; O'Carroll et al., 2018). It was noticed that this mutation is exactly located in the hydrophobic core of αC′–βD–αD screening pocket. Therefore, a mechanism of L252P mutation may have a significant impact on the αC′–βD–αD hydrophobic core discovered in this study. Additionally, it is the first time that this reliable and valid druggable pocket has been discovered and reported, which could be further employed in discovering inhibitors that block the polymerization of MyD88-TIR domain.

After flexibility exploration of the target protein MyD88, pocket detection, inhibitor screening, and multiple rounds of *in vivo* and *in vitro* activity tests, M20 stood out as a potential candidate to combat inflammatory diseases. M20, a novel MyD88 inhibitor with a pyrazolo [3,4-b] pyridine skeleton, which is totally different from previously reported inhibitors, such as peptidomimetic and its modified compounds. As a new type of MyD88 inhibitor, it broadens the structure developmental platform of MyD88 inhibitors. Computational chemistry, SPR, and co-immunoprecipitation assay have all demonstrated the crucial role of M20 in the inhibition of MyD88 homodimerization. Meanwhile, the release of the IL-6 inhibition rates of M20 in serum and BALF were more than 90% respectively, proving that it has great potential in combating with further MyD88-related diseases. In general, our work provides a lead compound for MyD88 inhibitors as well as a valuable novel drug candidate for LPS-stimulated inflammatory response, such as acute lung injury.

Moreover, the favorable compound shares a new pyrazolo [3,4-b] pyridine skeleton, which could be adopted as a lead compound for further chemical optimization. In the same batch of compounds, the five heterocyclic chain amide compounds M4, M7, M10, M13, and M19 also showed suitable anti-inflammatory effects. Compared with the core structure of M20, we speculate that the next step of structural modification of these candidates requires the introduction of larger heterocycles to enhance their biological activity. Overall, our data represent valuable progress in the development of MyD88 targeted drug candidates, which reveals the significant advancement in promoting the development and modification of more novel MyD88 inhibitors. Furthermore, considering that the co-crystallization structure of MyD88 and M20 has not yet been revealed, we conjecture that the preferred lead compound M20 will bind to the target protein in a more diverse form. Further studies on structural optimization and biological activities of the inhibitor are underway in our laboratory. We are committed to exploring this mechanism in more depth and will plan to report future findings.

### High Lights

1. A brand-new virtual screening pocket was discovered for the first time via the exploration of αC′ and αD flexible regions, and was successfully applied.
2. Computational chemistry, SPR, and co-immunoprecipitation verified that the best hit M20 could interact with MyD88 directly and inhibited the homodimerization.
3. M20 exhibited significant anti-inflammatory activities in LPS-induced responses, especially in ALI mice model mediated by sepsis.
4. The discovery of a new skeleton, pyrazolo [3,4-b] pyridine, revealed the significant implications in the development and transformation for MyD88 inhibitors.

## Data Availability

The datasets used and analyzed during the current study are available from the corresponding author on reasonable request.

## Abbreviations

DMEM, Dulbecco’s Modified Eagle’s Medium; ELISA, enzyme-linked immune sorbent assay; ERK, extracellular regulated protein kinases; FBS, fetal bovine serum; HA, influenza hemagglutinin epitope; IL-6, interleukin-6; ip, intra-peritoneally; iv, intravenous injection; JNK, c-Jun N-terminal kinase; MAPK, mitogen-activated protein kinase; qPCR, quantitative PCR; SDS-PAGE, sodium dodecyl sulfate-polyacrylamide gel electrophoresis; PBS, phosphate buffer saline; P38, P38MAPK; TNF-α, tumor necrosis factor-α.
