# Supplementary material for: Discovery of a Novel MyD88 Inhibitor M20 and Its Protection Against Sepsis-Mediated Acute Lung Injury
Source: Front Pharmacol. 2021 Nov 29;12:775117. doi: 10.3389/fphar.2021.775117 (PMC8666603; doi:10.3389/fphar.2021.775117)
Supplement: Supplementary file 1 [file DataSheet1.pdf]

Supplemental Material

Table S1 Basic drug information

No.	Molecule ID	Smiles	Docking Score (logP)	Purity	Relative molecular mass
M1	Z994229894	O=C(c1csc(-c2ncn[nH]2)n1)N1CCC[C@H]1Cc1ccccc1	2.7755	90+	339.424
M2	Z1279679613	Cc1cccc(-c2nnc(NC(=O)[C@@H]3COc4ccccc43)[nH]2)n1	2.28982	90+	321.34
M3	Z596022210	O=C(NCCc1cn2ccccc2n1)Nc1nnc(-c2ccccn2)s1	2.612	90+	365.422
M4	Z1198613251	O=C(Nc1ccn(-c2ccncc2)n1)[C@H]1CSc2ccccc2O1	2.7591	90+	338.392
M5	Z1145060898	Cc1cn(-c2ccccc2)nc1NC(=O)c1[nH]nc2ccccc12	3.30932	90+	317.352
M6	Z1274187005	CCc1ccc(-c2csc(NC(=O)c3cn(Cc4cccnc4)nn3)n2)cc1	3.6596	90+	390.472
M7	Z236785348	Cc1cccc(NC(=O)Cc2csc(COc3ccc(F)cc3)n2)n1	3.74582	90+	357.41
M8	Z976511916	O=C(Nc1nc(C(F)(F)F)n[nH]1)c1ccnc(Oc2ccccc2)c1	3.2631	90+	349.272
M9	Z1558844246	O=C(NCCC1CCCCC1)c1cccc(-c2ncn[nH]2)c1	3.1719	90+	298.39
M10	Z963870594	O=C(NCCC(=O)N1CCc2sccc2C1)Nc1ccccn1	2.2396	90+	330.413
M11	Z1262408789	O=C(Nc1nncs1)c1ccc(NCCc2ccccc2F)nc1	2.9791	90+	343.387
M12	Z1274265173	O=C(NCCc1ccc(F)cc1F)c1ccccc1Cn1cncn1	2.5771	90+	342.349
M13	Z1182458337	O=C(CN1CCN(C(=O)c2ncoc2-c2ccccc2)CC1)Nc1ccccc1	2.7381	90+	390.443
M14	Z295508412	Cc1nc(COc2ccccc2C(=O)Nc2ccc(-n3cccn3)nc2)no1	2.79002	90+	376.376
M15	Z911422760	O=C(CNC(=O)c1ccccc1)NCc1noc(-c2ccsc2)n1	1.8443	90+	342.38
M16	Z927583918	COc1ccc(NC(=O)CCc2c(C)nc(-c3ccccn3)[nH]c2=O)cn1	2.11522	90+	365.393
M17	Z31477621	O=C(CCC(=O)N1CCC(c2ccccc2)=N1)NCc1ccccc1	2.7196	90+	335.407
M18	Z1509741441	Cc1cc(NC(=O)CCN2CCN(c3nc(C)c(C)s3)CC2)no1	2.20716	90+	349.46
M19	Z821876118	COc1ccc2[nH]cc(C3CCN(CCC(=O)Nc4ccccc4F)CC3)c2c1	4.5238	90+	395.478
M20	Z223668298	O=C(CN1CCC(CCc2ccccc2)CC1)Nc1[nH]nc2ncccc12	3.2412	90+	363.465

Supplementary Table S1: Basic drug information. Compound source: ENAMINE; Agent: TOPSCIENCE and Accela ChemBio Co.,Ltd.

Table S2 Primers used for Quantitative real-time PCR			
**Gene**	**Species**	**FW (5′-3′)**	**RW (5′-3′)**
TNF-α	Mouse	CAGGGGCCACCACGCTCTTC	TTTGTGAGTGTGAGGGTCTGG
IL-6	Mouse	GAGGATACCACTCCCAACAGACC	AAGTGCATCATCGTTGTTCATACA
β-Actin	Mouse	CCGTGAAAAGATGACCCAGA	TACGACCAGAGGCATACAG

Supplementary Table S2: Primers used for Quantitative real-time PCR.
